# Comparison of the effects of erythropoietin and anakinra on functional recovery and gene expression in a traumatic brain injury model

**DOI:** 10.3389/fphar.2013.00129

**Published:** 2013-10-17

**Authors:** Gail D. Anderson, Todd C. Peterson, Cole Vonder Haar, Eric D. Kantor, Fred M. Farin, Theo K. Bammler, James W. MacDonald, Michael R. Hoane

**Affiliations:** ^1^Department of Pharmacy, University of WashingtonSeattle, WA, USA; ^2^Restorative Neuroscience Laboratory, Department of Psychology, Southern Illinois UniversityCarbondale, IL, USA; ^3^Department of Environmental and Occupational Health Sciences, University of WashingtonSeattle, WA, USA

**Keywords:** traumatic brain injury, recovery of function, cortical contusion impact, gene expression, erythropoietin, anakinra

## Abstract

The goal of this study was to compare the effects of two inflammatory modulators, erythropoietin (EPO) and anakinra, on functional recovery and brain gene expression following a cortical contusion impact (CCI) injury. Dosage regimens were designed to provide serum concentrations in the range obtained with clinically approved doses. Functional recovery was assessed using both motor and spatial learning tasks and neuropathological measurements conducted in the cortex and hippocampus. Microarray-based transcriptional profiling was used to determine the effect on gene expression at 24 h, 72 h, and 7 days post-CCI. Ingenuity Pathway Analysis was used to evaluate the effect on relevant functional categories. EPO and anakinra treatment resulted in significant changes in brain gene expression in the CCI model demonstrating acceptable brain penetration. At all three time points, EPO treatment resulted in significantly more differentially expressed genes than anakinra. For anakinra at 24 h and EPO at 24 h, 72 h, and 7 days, the genes in the top 3 functional categories were involved in cellular movement, inflammatory response and cell-to-cell signaling. For EPO, the majority of the genes in the top 10 canonical pathways identified were associated with inflammatory and immune signaling processes. This was true for anakinra only at 24 h post-traumatic brain injury (TBI). The immunomodulation effects of EPO and anakinra did not translate into positive effects on functional behavioral and lesion studies. Treatment with either EPO or anakinra failed to induce significant beneficial effects on recovery of function or produce any significant effects on the prevention of injury induced tissue loss at 30 days post-injury. In conclusion, treatment with EPO or anakinra resulted in significant effects on gene expression in the brain without affecting functional outcome. This suggests that targeting these inflammatory processes alone may not be sufficient for preventing secondary injuries after TBI.

## Introduction

Traumatic brain injury (TBI) is among the leading causes of acute and chronic disability in the United States. Out of the 1.7 million Americans that endure a TBI each year, over 50,000 die (Faul et al., 2010), making recovery of function a major public health issue. The first or primary injury relates to the initial injury caused by direct damage to the brain and only injury prevention will reduce the consequences of the primary injury. The second is indirect and progressive and is referred to as secondary injury. The etiology of the secondary cascade resulting from TBI is likely due to interrelated processes including mitochondrial energy failure, excessive generation of reactive oxygen species, activation of destructive enzymes, membrane disruption, neuronal death, thrombosis due to intravascular coagulation in small vessels, increased synaptic concentrations of excitatory amino acids, and activation of innate inflammatory responses (Wang et al., 2006; Jennings et al., 2008; McConeghy et al., 2012). Preventing the inflammatory response is considered a high potential target for neuroprotection after TBI (Schouten, 2007; Jennings et al., 2008). During secondary injury, early inflammation is triggered by multiple complex signals including, intracellular signaling, reactive oxygen and nitrogen species and loss of cellular homeostasis, all of which is mediated by microglia and astrocytes (Mathew et al., 1994; Juliet et al., 2008; Rock and Kono, 2008). An increase in the brain and cerebral spinal fluid pro-inflammatory cytokines [tumor necrosis factor_α_ (TNF_α_), interleukin (IL)-1β, IL-6], and anti-inflammatory cytokines [transforming growth factor (TGF-β, IL-4, IL-10)] occurs after injury in experimental TBI models and in patients with TBI (Morganti-Kossmann et al., 2001). Although the precise mechanisms are unknown, the immune response following inflammation directly induces apoptotic death and indirectly increases cell death by signaling cellular death programs (Shojo et al., 2010; Das et al., 2011).

The IL-1 family is a group of cytokines that are important for initiating and regulating immune and inflammatory responses. Animal models suggest that increasing levels of IL-1α and IL-1β exacerbate brain damage, while administering an IL 1 receptor antagonist (IL-1ra) reduces the infarct and improves recovery of function (Feurstein et al., 1997; Touzani et al., 1999). IL-1ra is a protein that in humans is encoded by the *IL1RN* gene and in rodent by the IL-1rn gene. Preventing IL-1 from binding to the IL-1 type receptor reduces inflammation (Feurstein et al., 1997; Touzani et al., 1999; So et al., 2007). There have been several experiments examining the role of IL-1ra in injury models other than ischemia, including subarachnoid hemorrhage, cryogenic injury, and excitotoxic injury. Following injury, IL-1ra reduced the number of nitric oxide synthase positive cells and improved both anatomical and functional outcomes (Jones et al., 2005). Elevation of IL-1ra after initiation of the inflammatory response is an important part of the auto-regulatory network controlling the inflammatory response (Bartfai et al., 2007). In patients with TBI, high concentrations of IL-1ra and high ratio of IL-1ra/IL-1β were association with better outcome (Hutchinson et al., 2007). IL-1ra reduces susceptibility to excitotoxic brain injury (Hagan et al., 1996), mediates convulsant actions of seizures in transgenic mice (Vezzani et al., 2004) and reduces damage following excitotoxic injury by attenuating neuronal insult and microglial activation in hippocampal slice cultures (Hailer et al., 2005) and in rodent models (Relton and Rothwell, 1992).

Altering this inflammatory pathway, preventing or reducing the inflammatory signal with an IL-1ra following TBI is a promising strategy in limiting the secondary injury. Erythropoietin (epoetin alfa; EPO) and anakinra, a recombinant form of non-glycosylated human IL-1 receptor antagonist are inflammatory modulators proposed as promising neuroprotective agents for the treatment of TBI. EPO, a hematopoietic growth factor that regulates red blood cell production also has significant pleiotropic effects (Boogaerts, 2006; Mammis et al., 2009). EPO has been shown to provide protection in a variety of models of neural damage, including focal ischemic stroke, experimental autoimmune encephalomyelitis, chemically-induced seizures, and blunt force trauma (Brines et al., 2000; Cerami et al., 2002). Subsequent studies have established beneficial effects of EPO administration on behavioral recovery from TBI (Brines et al., 2000; Cerami et al., 2002; Meng et al., 2011). EPO administration has been found to improve motor (Zhang et al., 2009), sensory (Xiong et al., 2008; Zhang et al., 2009), and cognitive function in experimental TBI (Lu et al., 2005; Yatsiv et al., 2005; Mahmood et al., 2007; Xiong et al., 2008; Zhang et al., 2009; Meng et al., 2011) and has also been shown to reduce lesion size (Xiong et al., 2008) and cell death after injury (Yatsiv et al., 2005; Zhang et al., 2009; Meng et al., 2011). Proposed mechanisms for the neuroprotective effect have included the ability of EPO to protect nerve cells from glutamate toxicity, reduce the immune response and inflammatory reaction, enhance nerve recovery, play a role in neurogenesis, prevent neuronal apoptosis, inhibit nitric oxide formation, and prevent oxidative stress (for a review, see Mammis et al., 2009). Research has shown that EPO can cross the blood brain barrier and affect neural tissues, increasing oxygenation to vulnerable tissues (Brines et al., 2000).

There is limited pre-clinical research evaluating treatment with IL-1ra in attenuating neuronal injury. IL-1ra has been shown to be a potential neuroprotective treatment in experimental studies in hippocampal slice cultures after excitotoxic damage (Hailer et al., 2005), in models of focal cerebral ischemia (Relton et al., 1996; Loddick et al., 1997), and in a fluid percussion brain injury model (Toulmond and Rothwell, 1995; Relton et al., 1996; Loddick et al., 1997; Lawrence et al., 1998; Sanderson et al., 1999; Hailer et al., 2005).

The objective of this study was to compare the effects of two inflammatory modulators, EPO and anakinra on functional recovery and gene expression following a unilateral controlled cortical impact (CCI) injury. This study is part of a project that assesses the effectiveness of several drugs on TBI and to identify the mechanism of action using microarray analysis. The ultimate goal is to use these data to design a multi-drug treatment therapy that will also be tested in our TBI model.

Prior to the CCI studies, single- and multiple-dose pharmacokinetic studies were performed in uninjured animals to determine the dose regimens needed to provide average serum concentrations in the range of the concentrations obtained clinically with FDA approved doses.

## Experimental procedures

### Animals and housing

Male, Sprague-Dawley rats (Harlan, Indianapolis, IN) approximately 3.5 months of age (mean body weight 350 g) were used in this study. All animal and surgical procedures were adhered to as described in the NIH Guide for the Care and Use of Laboratory Animals. The Southern Illinois University Institutional Animal Care and Use Committee (IACUC) and the University of Washington's IACUC reviewed and approved all experimental procedures. Animals were housed in a university-maintained, Association for Assessment and Accreditation of Laboratory Animal Care (AAALAC) accredited vivarium, with a 12-h light/dark schedule and a controlled environmental temperature of 22°C in standard housing cages with food and water available *ad libitum.*

### Pharmacokinetic studies

Male Sprague-Dawley rats with surgically implanted jugular vein catheters were obtained from Harlan Laboratories. For the single dose studies (*n* = 4/study), 100 mg/kg anakinra (Kineret™, Amgen, Thousand Oaks, CA) or 2500 IU/kg EPO (Procrit™, Amgen, Thousand Oaks, CA) were administered by subcutaneous (s.c.) or intraperitoneal injection (i.p.). Blood specimens were collected from the jugular catheter, immediately prior to the dose, and at 1, 2, 4, 6, 12, 24, and 30 h. Samples were collected in microtubes, separated using a centrifuge, and stored at −80°C until assayed. The terminal exponential rate constant (β) was used to determine elimination half-life (T_1/2_) as 0.693/β. Based on the single dose pharmacokinetics, the multiple doses were calculated to attain clinically relevant steady state concentrations. Targeted average concentrations of erythropoietin and anakinra were 5000–10,000 mIU/ml and 10–25 μg/mL, respectively, based on clinical studies in non-TBI patients receiving FDA approved doses. The initial dose was administered i.p. and subsequent doses administered s.c. every 12 h for 72 h. Blood was collected from a jugular catheter at 0, 1, 2 and then immediately before and 1 h after injections for 72 h. Serum concentrations were determined using commercially available ELISA kits (R&D Systems, Minneapolis, MN).

### Experimental injury model

All surgeries were performed under aseptic conditions. The CCI injury model utilized in the present study was based on previous studies (Anderson et al., 2011, 2013; Peterson et al., 2012). Animals were anesthetized using a mixture of isoflurane (2–4%) and oxygen (0.8 L/min). Once the animal was no longer responsive (no ocular or pedal reflexes) the head was shaved and scrubbed with 70% alcohol followed by betadine and placed into a stereotaxic device. A midline incision was made through the skin and the underlying fascia. A circular craniotomy (5.0 mm) was centered 2.4 mm posterior to and 2.4 mm lateral (left) to bregma. The contusion injury was created with a sterile stainless steel impactor tip (4.0 mm in diameter) that was attached to the Benchmark™ stereotaxic impactor (www.myneurolab.com, St. Louis, MO). The injury was induced with an impact speed of 3.0 m/s and depth of 2.5 mm, coming in contact with the dura for 0.5 s before retraction. To maintain normal body temperature (37°C) during surgery and recovery the rats were placed on a warm water recycling bed and pump system (EZ Anesthesia, Palmer, PA). Rats receiving sham surgeries underwent identical surgical preparation as injured animals, with the exception of the impact (received craniotomies), were sutured, and then transferred to recovery. The design was completely counter-balanced to ensure that all animals received the same number of injections, regardless of treatment condition.

### Drug administration

Animals were randomly assigned to one of four groups: (a) Sham, (b) CCI-injured erythropoietin (EPO) 2500 IU/kg (c) CCI-injured anakinra at 100 mg/kg or (d) CCI-injured vehicle. Based on the results of the pharmacokinetic studies, in order to obtain peaks concentration within 1–2 h, the initial dose was administered by i.p. injection and subsequent injections were administered by s.c. Doses were administered 2 h, 12 h, 24 h, 36 h, 48 h, 60 h, and 72 h after the CCI injury. Both EPO and anakinra were diluted into 0.9% sterile saline (Sigma Aldrich Co, St. Louis, MO) such that the amount of fluid per kg body weight was the same. Blood samples (750 μ L) were collected in microtubes from the tail vein 1 h after the final injection, separated using a microcentrifuge, and stored at −80°C until assayed. For the functional behavioral studies, a power analysis determined that group sizes of nine results in a power score >0.85 to detect significant differences between the groups.

### Gene expression studies

At specified time point's post-CCI (24 h, 72 h, and 7 days) 5 intact sham animals (no craniotomy) and 5 animals in each treatment group were overdosed with a mixture of CO_2_ (80%) and O_2_ (20%). The rats were then decapitated, a cardiac blood sample collected and brains were rapidly extracted. To maintain quality control and to assure that all of the brains were injured, each brain was assigned a rating score (1 = no visual sign of trauma; 2 = bruised and swollen cortex; 3 = no remaining cortex or extensive damage) (see Figure 1 in Anderson et al., 2011). Only brains with a score of 2 were used in the subsequent analyses. The brain was then cut into a 4.0 mm coronal slab containing the injury site in a brain matrix (Braintree Scientific, Inc., Braintree, MA) and placed onto an RNAase free cold plate. A 5.0 mm biopsy punch was used to collect the injury site and surrounding cortical tissue (Hoane et al., 2006; Anderson et al., 2011). The tissue punch included all injured cortical tissue and a small strip of pericontusional tissue, with the ventral extent of the punch extending to the corpus callosum. Tissue punches were placed into microcentrifuge tubes, snap frozen and then stored at −80°C. All samples were shipped by overnight carrier to the University of Washington on dry ice.

Processing of the RNA samples, hybridization to the microarray, scanning with the AffymetrixGeneChip® 3000 scanner and univariate analysis of the data using various Bioconductor packages were performed as previously described (Anderson et al., 2011, 2013). Venn diagrams were generated with the Bioconductor limma package. Ingenuity Pathway Analysis (IPA) (Build 131235; Version 11904312; Database Status 02.20.2012; www.ingenuity.com) was used to analyze differentially expressed genes (>1.5-fold up or down-regulated, *p* < 0.05) using the Core Analysis feature. IPA is a commercial tool that is based on a proprietary database (www.ingenuity.com/) to facilitate the identification of biological themes in microarray gene expression data. IPA maps the rat genes to their human orthologs and uses the human orthologs in its pathway analysis. IPA uses a Right-tailed Fisher's exact test to determine the probability that the number of genes annotated to a given biological function, canonical pathway or transcriptional network in the set of significant genes is due to chance alone. The validation of the data obtained with the microarrays was performed using fluorogenic 5′-nuclease-based assay and quantitative RT-PCR as previously described (Anderson et al., 2011, 2013). The RT-PCR data was normalized to the housekeeping gene, β-actin.

### Functional behavioral studies

#### Sensorimotor/motor assessment

***Locomotor placing task.***To assess recovery of coordinated, locomotor limb movement, this test was administered on days 2, 4, 6, 8, and 10 post-CCI following methods outlined in previous studies (Hoane et al., 2008b; Goffus et al., 2010; Peterson et al., 2012). Rats were pre-tested to establish baseline levels for 2 days before surgery. On each test day, the rat was placed on an elevated grid floor (56.0 × 54.0 cm) with openings measuring 3.2 × 3.2 cm in size and allowed to freely explore for 120 s. A “foot-fault” occurred when a rat inaccurately placed a limb through one of these openings. Total movement on the grid as measured by the number of lines crossed as well as foot-faults for each limb was recorded. Rats were administered one trial each test day. The total number of foot-faults for the limb contralateral to the injury was the primary dependent variable of interest. The following equation was used to calculate the foot faults as a function of total movement on the grid: (Right sforelimb faults - Left forelimb faults)/lines crossed.

***Rotorod.*** Automated ROTOR-ROD™ (San Diego Instruments, San Diego, CA) testing was performed on post-injury days 8–12 to assess gross motor coordination. Two days of training occurred prior to surgery to establish a pre-injury baseline on the rotorod. Animals were placed on the rotating cylinder (7 cm diameter) against the rotation and the latency to fall (1.3 m) was recorded. On the first day of training, animals received one training trial where they were placed on the cylinder, which was stationary, and replaced when they fell, until they were able to remain on the cylinder for 60 consecutive seconds so that they become acclimated to the device. Following this trial and on all other days of training, the trial was terminated and the latency was recorded when the animal fell off of the cylinder. The initial speed of 5 rpm was maintained for 15 s to allow experimenters to load the rat. Following this 15 s, the speed of the rotation increased from 5 to 50 rpm over the course of 300 s (acceleration rate of 0.15 rpm/s). An infrared beam sensor located below the cylinder recorded the latency for the animal to fall. Each animal received four trials each day with a 15 min inter-trial interval.

#### Cognitive assessment

***Morris water maze (MWM)—reference memory task.*** All animals were tested in the MWM using a reference memory paradigm which has been widely utilized to assess cognitive performance following TBI (Lindner et al., 1998; Hoane et al., 2003, 2006; Vonder Haar et al., 2011; Peterson et al., 2012). The apparatus consisted of a circular, 180 cm diameter blue plastic pool partially filled with water (22°C) to a depth of approximately 32 cm. A clear plastic platform (10 × 10 cm) was submerged approximately 2 cm below the surface located in the northeast quadrant of the pool for the entirety of the reference memory testing sessions. The animal's progress on the task was evaluated by a video camera affixed above the pool and this data was processed using computer software SMART (San Diego Instruments, San Diego, CA). Animals were assessed on the acquisition of a reference memory task beginning on day 14 post-CCI and were tested for 4 subsequent days. On each testing day, animals received four trials to locate the submerged platform in the pool, starting at one of four release points in random order. The trial was terminated when the rat reached the submerged platform in the northeast quadrant or when 90 s had elapsed. Latencies were recorded, averaged and used as the main dependent variable. If the rat did not find the platform within the 90 s, it was guided to the platform and remained on it for at least 10 s. Each rat remained on the platform for 10 s, after which it was placed in a warm holding cage for at least 15 min before the next trial.

***MWM—working memory task.*** Animals were tested on days 21–23 post-CCI using established methods (Lindner et al., 1998; Hoane et al., 2003, 2006; Vonder Haar et al., 2011; Peterson et al., 2012). The procedure was the same as above, except the platform was submerged at the center of a new randomly chosen quadrant (south-west, north-west, and south-east) each day. Each animal was given four trials per day, starting from one of four randomly chosen release points (inter-trial interval was 15 min). The first trial on each of these days was considered an information trial and was not included in subsequent analyses; the latencies from the last three trials were averaged to form a score for the day. Each trial was terminated when the animal located the platform, or when 90 s had elapsed. Animals not reaching the platform were guided to the platform after the trial ended.

#### Lesion analysis

At 30 days post-CCI, the rats were euthanized with Euthasol (Virbac Animal Health; 0.3 mL i.p.) and transcardially perfused with 0.9% phosphate-buffered saline (PBS), followed by 10% phosphate buffered formalin (PBF). Brains were post-fixed in PBF for 2 days following removal from the cranium. A 30% sucrose solution was used to cryopreserve the brains 3 days prior to frozen sectioning. Serial, coronal sections (40 μm thickness) were sliced using a sliding microtome on a frozen stage and collected into a cryopreservative solution and stored at −20°C. A series of sections were brush mounted on gelatin-subbed microscope slides, stained with cresyl violet, dehydrated, and cover slipped. The extent of the lesion was analyzed with an Olympus microscope (BX-51) and an Olympus 13.5 megapixel digital camera (DP-70). Images of sections throughout the extent of the injury coordinates were captured using the digital capturing system and area measures of the lesioned tissue were determined using the ImageJ software package (1.43 u, NIH). The Calvalieri method was used to calculate the volumes of the ipsilateral cortex and the contralateral cortex (Coggeshall, 1992; Vonder Haar et al., 2011; Peterson et al., 2012). Four stereotaxic coordinates throughout the lesion, at approximately −0.80, −1.80, −2.80, and −3.80 mm relative to bregma, were selected for lesion analysis. The number of sections and the section thickness (40 μm) were multiplied by the mean area of the remaining cortex. The extent of cortical injury was measured by calculating the percent reduction in the injured ipsilateral cortex compared to the contralateral cortex at each level using the formula: [1 − (ipsi/contra) × 100] and which has been shown to be sensitive enough to detect treatment-induced reductions in injury size (Hoane et al., 2004, 2006, 2008a, 2009).

#### Data analysis

Behavioral data was analyzed using a mixed model factorial ANOVA and histological data was analyzed using a one-way between-subjects ANOVA (SPSS v. 15 for Windows). The between factor of Treatment (EPO-treated, anakinra-treated, vehicle-injured, and sham) and the within-group factor was day of testing. Both the main effects and the interaction effects were considered. Huynh-Feldt corrections and Tukey's Honestly Significant Different test (Tukey's *HSD*) were used to control for Type I error in the repeated measures and *post-hoc* means comparison, respectively. A significant level of *p* = 0.05 was used for all statistical analyses. A One-Way ANOVA was completed where the between-subject factor of Treatment (EPO-treated, anakinra-treated, vehicle-injured, and sham) was used to analyze the anatomical data. Tukey's HSD was used to control for Type I error and a significant level of *p* ≤ 0.05 was used for all statistical analyses. Inter-rater reliability measures were evaluated using the bivariate correlation of scores obtained between the two raters. A power analysis indicated that *n* = 10 for the behavioral studies would give a power rating of 90% and that *n* =5 for the pharmacokinetic and molecular studies would also allow for detection of significant differences (Anderson et al., 2013). The evaluators were blinded to the treatment assignment.

## Results

### Anakinra and erythropoietin pharmacokinetics

**Figure 1:**
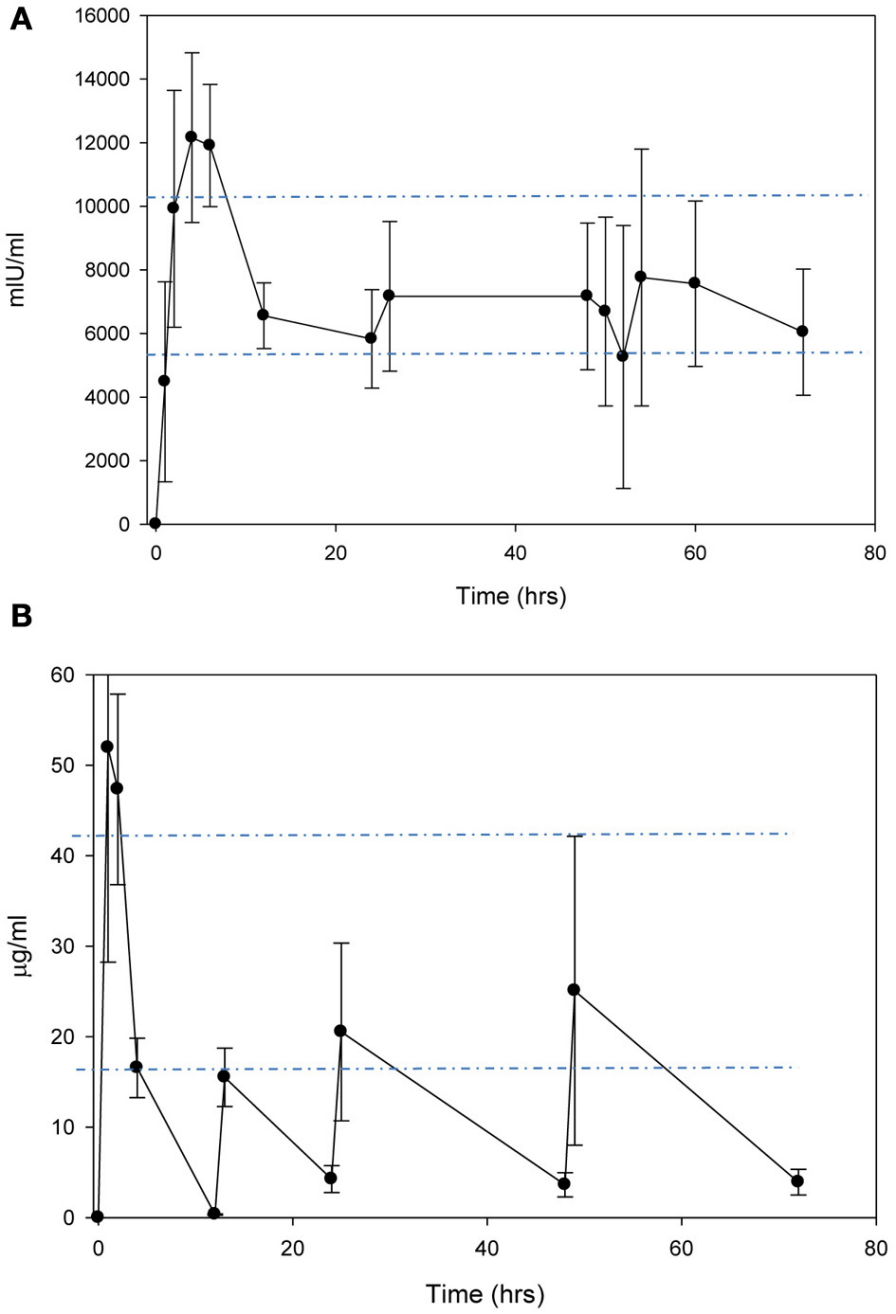
**Concentration-time curves after administration of (A) EPO 2500 mg/kg i.p**. for the first dose, followed by s.c. every 12 h and **(B)** anakinra 100 mg/kg i.p. for the first dose, followed by s.c. every 12 h.

In the non-injured animals, a single dose i.p. or s.c. injection of EPO 2500 IU/kg, resulted in peak concentrations of 4481 ± 1436 mIU/mL and 2086 ± 201 mIU/mL at 2 and 24 h, respectively. Due to the prolonged absorption from the s.c. dose, the apparent T_1/2_ (mean ± standard deviation) was 16.9 ± 2.6 h compared to 8.9 ± 0.8 h after i.p. Anakinra 100 mg/kg administered i.p. produced peak concentrations of 62.1 ± 31.7 μ g/mL at 1 h post-dose with a T_1/2_ of 1.8 ± 0.4 h. When administered s.c., anakinra peak concentrations of 23.5 ± 2.9 μ g/mL occurred 2 h post-dose. Therefore, multiple doses, with the initial dose by i.p. followed by s.c. every 12 h, resulted in clinically relevant EPO concentrations with minimal fluctuations with peak and trough concentrations of 13,375 ± 1411 mIU/mL and 13,395 ± 1436 mIU/mL, respectively (Figure 1A). Multiple doses of anakinra produced an initial peak concentration of 51.9 ± 23.6 μ g/mL and then average plasma concentrations of 20.3 ± 6.9 μ g/mL (Figure 1B). In the functional behavioral and gene expression studies, the EPO and anakinra serum concentrations were 9990 ± 1054 mIU/mL and 9127 ± 944 mIU/mL and 40 ± 7 μg/mL and 28 ± 14 μg/mL when sampled 72 h post-CC, respectively.

### Functional behavioral study

**Figure 2:**
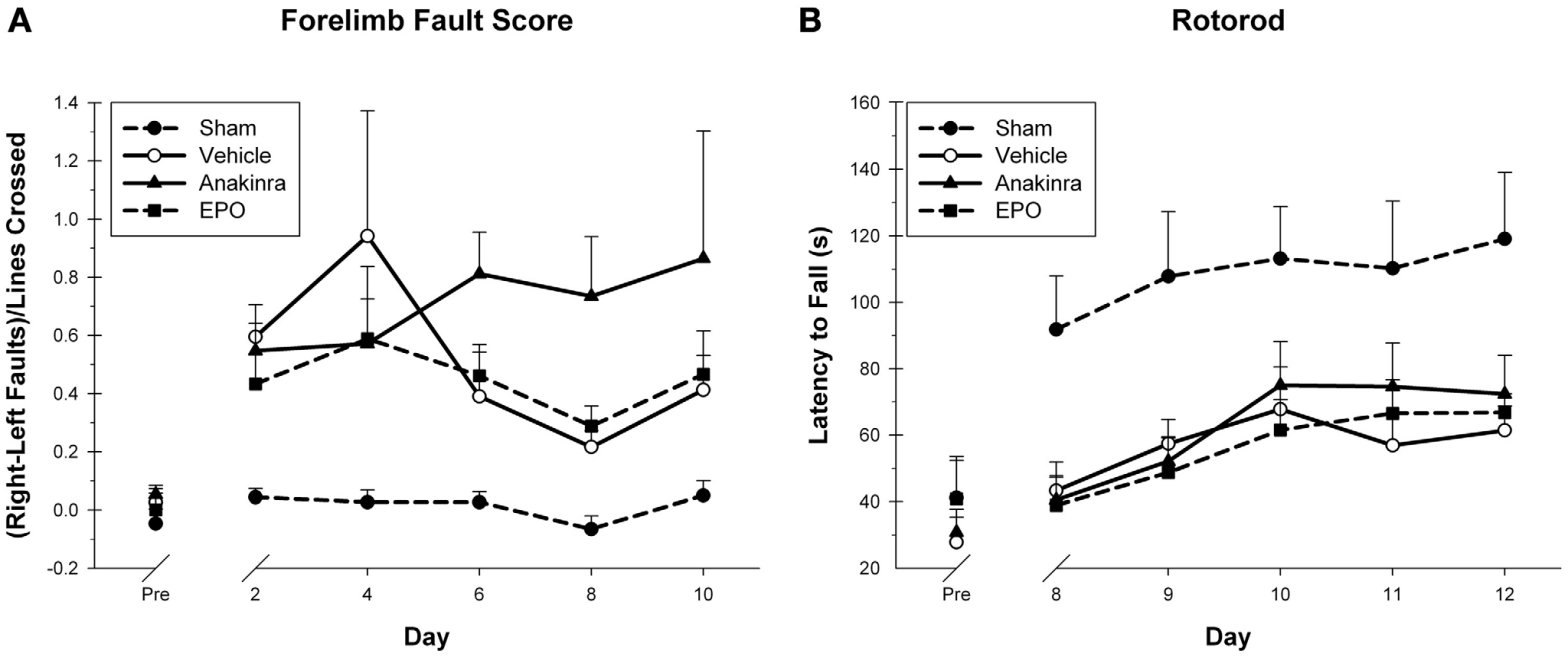
**Sensorimotor Assessment. (A)** The Locomotor Placing task showing the average fault scores (+SEM) for days 2, 4, 6, 8, and 10 post-CCI. No significant differences between the treated animals and vehicle were found. A trend toward worsening was seen in the anakinra-treated group. **(B)** The Rotorod test showing the average latency to fall (+SEM) off of the rotating cylinder for day's 8–12 post-CCI. No significant differences between the treated animals and vehicle were found.

In the Locomotor placing task, foot faults score was used as the primary dependent variable (Figure 2A). The within-subjects factor of Day (2, 4 6, 8, and 10) and the between-subjects factor of Treatment (EPO-treated, anakinra-treated, vehicle-injured, and sham) were significant. The interaction of Day × Treatment was significant [*F*_(12, 168)_ = 2.32, *p* < 0.05]. Both the main effect of Day [*F*_(4, 168)_ = 4.39, *p* < 0.05] and Treatment [*F*_(3, 42)_ = 106.71, *p* < 0.001] were significant. The sham group performed significantly better than the EPO-treated group [*HSD*_(19)_ = 3.37, *p* < 0.05], the anakinra-treated group [*HSD*_(19)_ = 4.09, *p* < 0.05], and the vehicle-injured group [*HSD*_(22)_ = 4.19, *p* < 0.05]. There were no significant differences between either of the drug treatment groups and the vehicle-injured group.

On the Rotor-Rod, the latency to fall off the rotating cylinder was averaged over the four trials for each testing day and used as the dependent variable to measure gross motor function with the within-subjects factor of Day (8–12) and the between-subjects factor of Treatment (EPO-treated, anakinra-treated, vehicle-injured, and sham). The interaction of Day × Treatment was not significant [*F*_(11.68, 163.50)_ = 8.28, *p* = 0.830]. Both the main effect of Day [*F*_(3.893, 163.5)_ = 10.75, *p* < 0.001] and Treatment [*F*_(3, 42)_ = 3.98, *p* < 0.05] were significant, see Figure 2B. The sham group performed significantly better than the EPO-treated group [*HSD*_(19)_ = 41.23, *p* < 0.05], the anakinra-treated group [*HSD*_(19)_ = 37.59, *p* < 0.05], and the vehicle-injured group [*HSD*_(22)_ = 41.68, *p* < 0.05]. There were no significant differences between either of the drug treatment groups and the vehicle-injured group.

**Figure 3:**
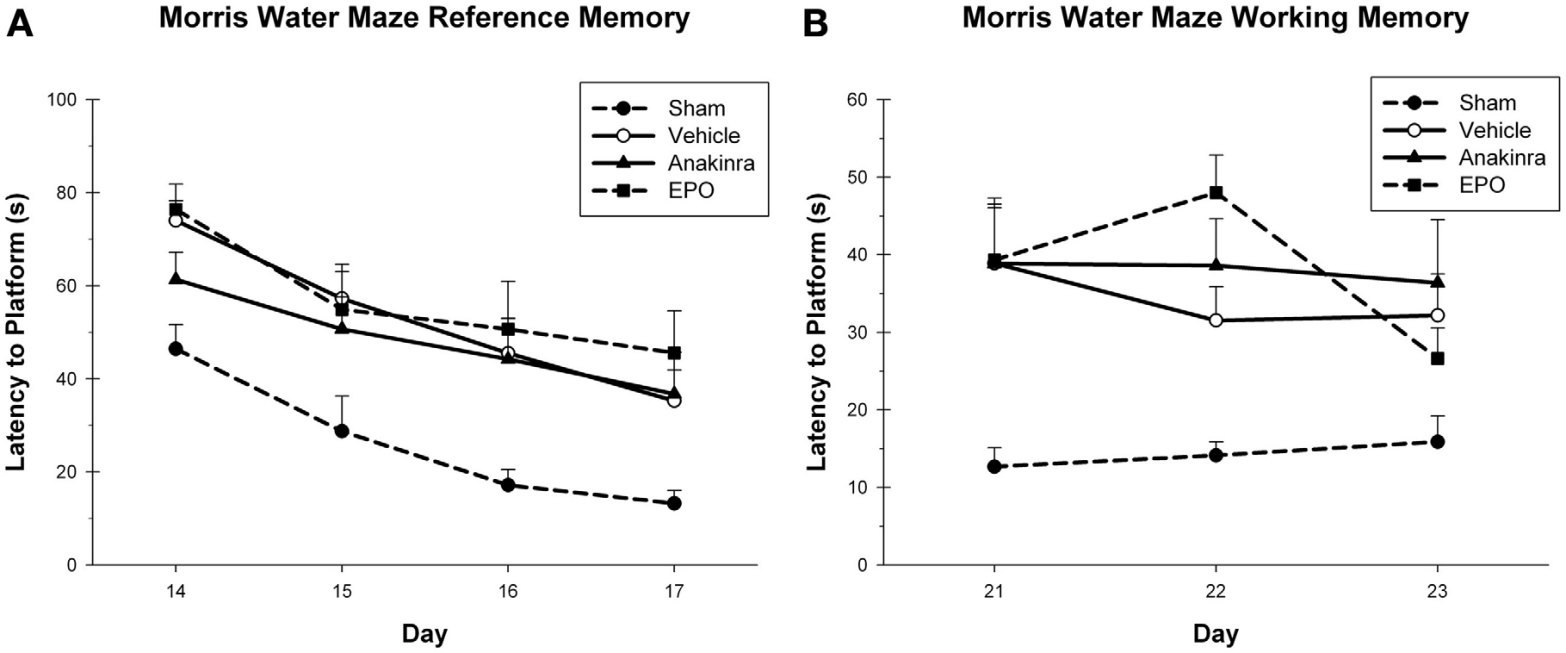
**Cognitive assessment. (A)** The MWM reference memory task showing the average latency (+SEM) to reach the platform on day's 14–17 post-CCI. No significant differences between the treated animals and vehicle were found. **(B)** The MWM working memory task showing the average latency (+SEM) to reach the platform on day's 21–23 post-CCI. No significant differences between the treated animals and vehicle were found.

In the reference memory task, the latency to reach the platform was averaged over the four trials for each testing day during reference memory acquisition (Figure 3A), with the within-subjects factor of Day (14–17) and the between-subjects factor of Treatment (EPO-treated, anakinra-treated, vehicle-injured, and sham). The interaction of Day × Treatment was not significant [*F*_(9, 120)_ = 0.50, *p* = 0.87]. Both the main effect of Day [*F*_(3, 120)_ = 35.14, *p* < 0.001] and Treatment [*F*_(3, 40)_ = 5.03, *p* < 0.05] were significant. The sham group performed significantly better than the EPO-treated group [*HSD*_(19)_ = 27.08, *p* < 0.05], the anakinra-treated group [*HSD*_(19)_ = 20.64, *p* < 0.05], and the vehicle-injured group, [*HSD*_(22)_ = 21.11, *p* < 0.05]. There were no significant differences between either of the drug treatment groups and the vehicle-injured group.

In the working memory task, the latency to reach the platform was averaged over the last three trials for each testing day during working memory acquisition, with the within-subjects factor of Day (21–23) and the between-subjects factor of Treatment (EPO-treated, anakinra-treated, vehicle-injured, and sham), see Figure 3B. The interaction of Day × Treatment was not significant [*F*_(6, 80)_ = 1.26, *p* = 0.291]. The main effect of Day [*F*_(2, 80)_ = 2.07, *p* = 0.131] was not significant, but the main effect of Treatment [*F*_(3, 40)_ = 9.57, *p* < 0.001] was significant. The sham group performed significantly better than the EPO-treated group [*HSD*_(19)_ = 22.13, *p* < 0.001], the anakinra-treated group [*HSD*_(19)_ = 22.89, *p* < 0.001], and the vehicle-injured group [*HSD*_(22)_ = 18.74, *p* < 0.001]. There were no significant differences between either of the drug treatment groups and the vehicle-injured group.

### Lesion analysis

**Figure 4:**
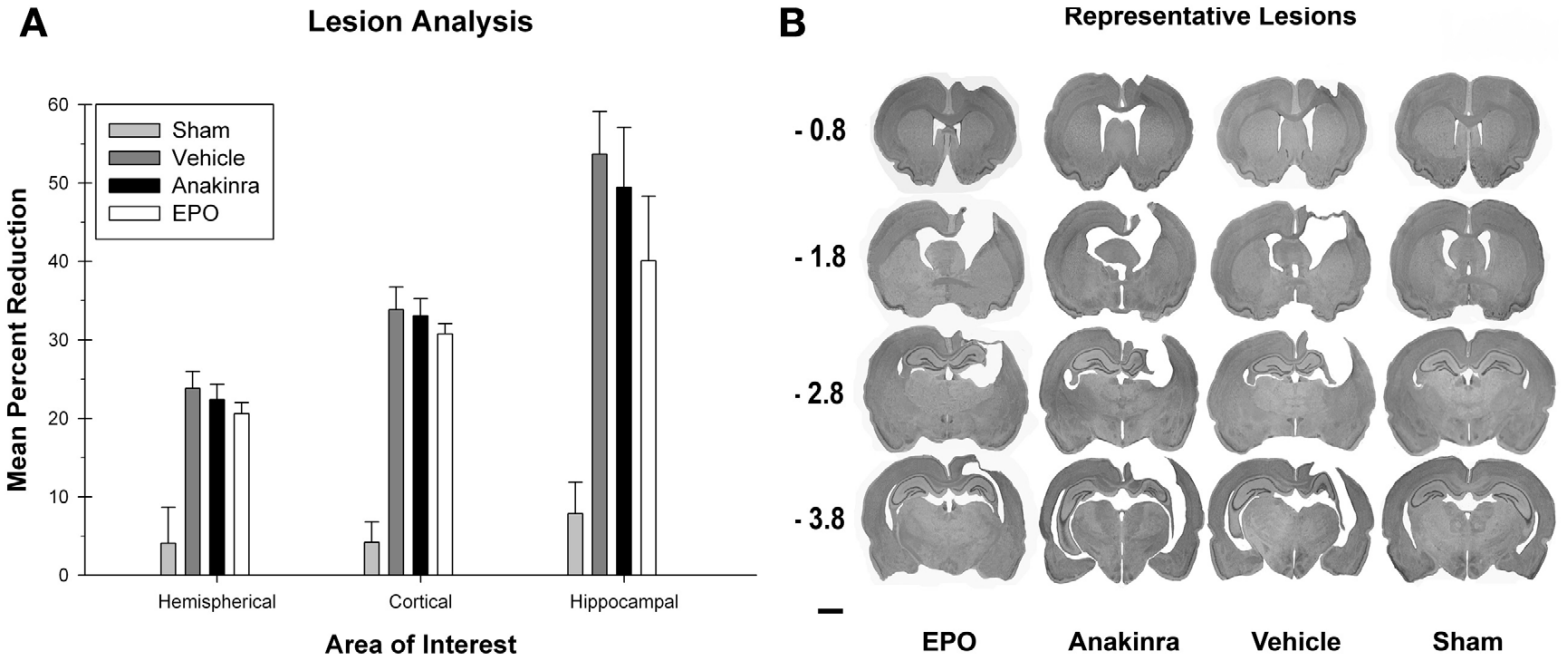
**Lesion Analysis. (A)** The average (+SEM) percent reduction in hemispherical, cortical, and hippocampal volumes between the ipsilateral and contralateral sides of the injury. No significant differences between the treated animals and vehicle were found. **(B)** Representative images of cresyl violet stained tissue throughout the injury coordinates; −0.08 mm, −1.8 mm, −2.8 mm, and −3.8 mm, relative to bregma. Scale bar = 2.0 mm.

The ratio of lesion volume in the cortices, hippocampus, and hemisphere for each group were each compared with a one-way ANOVA [Group (EPO-treated, anakinra-treated, vehicle-injured, and sham)], see Figure 4A. There was a significant difference between the groups [*F*_(3, 35)_ = 42.13, *p* < 0.001] for lesion volume in the cortices. *Post-hoc* analysis showed that the sham group had significantly more cortical volume compared to the EPO-treated group [*HSD*_(19)_ = 29.56, *p* < 0.001], the anakinra-treated group [*HSD*_(19)_ = 28.87, *p* < 0.001], and the vehicle group [*HSD*_(22)_ = 29.65, *p* < 0.001]. There were no significant differences between either of the drug treatment groups and the vehicle-injured group.

There was a significant difference between the groups [*F*_(3, 35)_ = 11.19, *p* < 0.001] for lesion volume in the hippocampus. *Post-hoc* analysis showed that the sham group had significantly more hippocampal volume compared to the EPO-treated group [*HSD*_(19)_ = 32.22, *p* < 0.05], the anakinra-treated group [*HSD*_(19)_ = 41.59, *p* < 0.001], and the vehicle group [*HSD*_(22)_ = 45.82, *p* < 0.001]. There were no significant differences between either of the drug treatment groups and the vehicle-injured group.

There was a significant difference between the groups [*F*_(3, 35)_ = 11.9, *p* < 0.001] for lesion volume in the hemispheres. *Post-hoc* analysis showed that the sham group had significantly more hemispherical volume compared to the EPO-treated group [*HSD*_(19)_ = 16.55, *p* < 0.001], the anakinra-treated group [*HSD*_(19)_ = 18.35, *p* < 0.001], and the vehicle group [*HSD*_(22)_ = 19.75, *p* < 0.001], see Figure 4. There were no significant differences between either of the drug treatment groups and the vehicle-injured group. See Figure 4B for representative lesion images for the experimental groups.

### Gene expression

**Figure 5:**
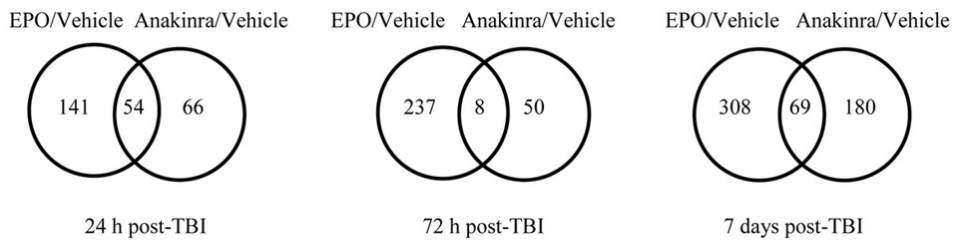
**The Venn diagrams show the number of genes whose expression was up or down regulated more than 1.5-fold (*p* < 0.05) in the EPO/Vehicle and Anakinra/Vehicle contrasts at the 24 h, 72 h, and 7 day time points**. Venn diagrams were generated with the Bioconductorlimma package.

The microarray data passed all the standard and advanced quality control metrics. The number of differentially expressed genes (>1.5-fold change, *p* < 0.05) at 24 h, 72 h and 7 days are presented in Table 1. The vehicle to sham comparison reflects the effect of the TBI without treatment relative to sham controls. The EPO or anakinra (CCI animals that received treatment) to vehicle (CCI animals that received vehicle) comparison evaluates the effect of treatment on gene expression in the context of TBI. Both EPO and anakinra treatments resulted in significant changes in brain gene expression in the CCI model demonstrating acceptable brain penetration. At all three time points, EPO treatment resulted in significantly more differentially expressed genes than anakinra. At 7 days post-injury (4 days after the last dose of EPO or anakinra), 337 genes were differentially expressed by EPO with 96% (322/337) showing increased expression. Similarly, at 7 day post injury, 209 gene were differentially expressed by anakinra with 85% of genes (178/209) showing increased expression. The Venn diagrams in Figure 5 show the number of differentially expressed genes (1.5-fold, *p* < 0.05) that are unique for each of the EPO/vehicle and ankinra/vehicle contrasts, as well as the number of genes that are shared between these contrasts for each of the three time points. At 24 h and 7 days post-TBI, approximately a quarter of the genes differentially expressed with EPO treatment also were affected by anakinra treatment. At 72 h post-TBI, only 8 (3%) of the 58 genes differentially expressed by anakinra treatment compared to vehicle were also differentially expressed with EPO treatment.

**Table 1:** **The number of differentially expressed genes probes (>1.5-fold up or down, *p* < 0.05)**.

	**24 h**			**72 h**			**7 days**		
	**Down**	**Up**	**Total**	**Down**	**Up**	**Total**	**Down**	**Up**	**Total**
**Vehicle/Sham**	1014	1457	2471	1330	1956	3286	318	1104	1422
**EPO/Vehicle**	137	58	195	178	47	245	15	322	337
**Anakinra/Vehicle**	103	17	120	33	25	58	31	178	209

Ingenuity Pathway Analysis (IPA) was used to facilitate the identification of biological themes in the microarray data. IPA shifts the emphasis from the evaluation of single genes to an evaluation of molecular pathways, networks and biological functions. Functional categories are identified by molecular and cellular functions. For anakinra at 24 h and EPO at all three times points, the regulated genes in the top 3 functional categories were involved in cellular movement (primarily inflammatory cells), inflammatory response and cell-to-cell signaling. For anakinra, at 7 d post-TBI, the top functional categories were molecular transport, organismal injury and abnormalities and carbohydrate metabolism. Differentially expressed genes of interest were selected and their specific fold changes in expression for the contrast EPO/vehicle, anakinra/vehicle and vehicle/shame are presented in Table 2. Of note, IL 1b, IL 1 receptor, type II, IL 1 receptor antagonist (IL-1rn), several chemokines and chemokine receptors and nitric oxide synthase were significantly increased by TBI and decreased by both EPO and anakinra. Specifically, administration of anakinra decreased gene expression of endogenous IL-1rn only at the 24 h with no effect at 72 h or 7 days and EPO decreased the gene expression of IL-1rn at both 24 and 72 h post-injury.

**Table 2:** **The effect of Erythropoietin, Anakinra and TBI (vehicle/sham) on genes of interest (1.5 fold change, *p* < 0.05)**.

**Affymetrix ID**	**Gene symbol**	**Genes**	**EPO vehicle**	**Anakinra vehicle**	**Vehicle sham**
**24 H**					
10775968	Alb	Albumin	1.91	n.s.	0.50
10911380	Aldh1a2	Aldehyde dehydrogenase 1 family, member A2	1.97	n.s.	n.s.
10744425	Alox15	Arachidonate 15-lipoxygenase	3.76	1.95	n.s.
10821016	Ccnb1	Cyclin B1	1.50	n.s.	n.s.
10717195	Ccnb2	Cyclin B2	1.53	n.s.	n.s.
10921163	Ccr1	Chemokine (C-C motif) receptor 1	0.49	0.59	9.78
10829761	Cdk1	Cyclin-dependent kinase 1	1.50	n.s.	2.36
10775896	Cxcl3	Chemokine (C-X-C motif) ligand 2	0.48	n.s.	11.92
10924245	Cxcr2	Chemokines (C-X-C motif) receptor 2	0.49	0.47	18.82
10825153	Fcgr1a	Fc fragment of IgG, high affinity Ia, receptor	0.66	n.s.	7.36
10755148	Fetub	Fetuin B	n.s.	0.64	2.48
10926967	Gsta2	Glutathione-S-transferase alpha 2	1.56	n.s.	0.42
10849841	IL1b	Interleukin 1 beta	0.48	0.59	6.82
10922816	IL1r2	Interleukin 1 receptor, type II	0.53	0.64	7.10
10834109	IL1rn	Interleukin 1 receptor antagonist	0.46	0.59	17.23
10922882	IL18rap	Interleukin 18 receptor accessory protein	0.64	0.66	2.38
10907689	Itga5	Integrin, alpha 5	0.66	n.s.	4.63
10711299	Itgax	Integrin, alpha X	0.61	0.44	4.35
10810736	Lcat	Lecithin cholesterol acyltransferase	1.55	n.s.	0.44
10736312	Nos2	Nitric oxide synthase 2, inducible	0.42	0.51	7.71
10873341	Pla2g2a	Phospholipase A2, group IIA	n.s.	1.77	2.23
10708214	Prc1	Protein regulator of cytokinesis 1	1.52	1.64	1.54
10835817	Ptgs1	Prostaglandin-endoperoxide synthase 1	n.s.	1.52	n.s.
10764551	Ptgs2	Prostaglandin-endoperoxide synthase 2	0.56	n.s.	2.67
10742194	Pttg1	Pituitary tumor-transforming 1	1.57	n.s.	n.s.
10738130	Rara	Retinoic acid receptor, alpha	0.66	n.s.	1.83
10786108	Rarb	Retinoic acid receptor, beta	1.94	n.s.	0.25
10827686	RT1-M6-1	RT1 class I, locus M6, gene 1	0.46	n.s.	2.13
10827691	RT1-M6-2	RT1 class I, locus M6, gene 2	0.48	n.s.	2.21
10817071	S100a8	S100 calcium binding protein A8	n.s.	0.66	3.28
10934608	Tir13	Toll-like receptor 13	0.64	0.17	6.31
10770577	Tgfb2	Transforming growth factor, beta 2	0.63	n.s.	n.s.
10746976	Top2a	Topoisomerase (DNA) II alpha	1.50	n.s.	3.26
**72 H**					
10885393	Akap5	A kinase (PRKA) anchor protein 5	1.54	n.s.	0.57
10932912	Alas2	Aminolevulinate, delta-, synthase 2	2.96	1.87	n.s.
10744425	Alox15	Arachidonate 15-lipoxygenase	14.84	1.75	n.s.
10736697	Ccl2	Chemokines (C-C motif) ligand 2	0.66	n.s.	45.91
10736702	Ccl7	Chemokines (C-C motif) ligand 7	0.52	n.s.	9.29
10858566	Clec4a2	C-type lectin domain family 4, member A2	0.61	n.s.	7.66
10866019	Clec7a	C-type lectin domain family 7, member A	0.59	n.s.	30.82
10914614	Ccr2	Chemokine (C-C motif) receptor 2	0.51	n.s.	11.00
10865585	Cd4	Cd4 molecule	0.53	n.s.	2.86
10856274	Cd8a	CD8a molecule	0.57	n.s.	5.90
10856265	Cd8b	CD8b molecule	0.56	n.s.	5.85
10802013	Cd72	Cd74 molecule, major histocompatibility complex, class II invariant chain	0.64	n.s.	3.93
10897428	Csf2rb	Colony stimulating factor 2 receptor, beta, low-affinity (granulocyte-macrophage)	0.60	n.s.	10.88
10767373	Cxcr4	Chemokine (C-X-C motif) receptor 4	0.63	n.s.	2.65
10811571	Cyba	Cytochrome b-245, alpha polypeptide	0.66	n.s.	3.95
10936899	Cybb	Cytochrome b-245, beta polypeptide	0.65	n.s.	17.68
10732652	Dusp1	Dual specificity phosphatase 1	n.s.	1.51	0.40
10828344	HLA-DRA^a^	Major histocompatibility complex, class II, DR alpha	0.55	0.65	7.18
10831657	HLA-DRB1^a^	Major histocompatibility complex, class II, DR beta 1	0.63	0.69	2.50
10828351	HLA-DQA1^a^	Major histocompatibility complex, class II, DQ alpha 1	0.60	0.71	3.92
10767518	Ikbke	Inhibitor of kappa light polypeptide gene enhancer in B-cells, kinase epsilon	0.66	n.s.	2.30
10834109	ILr1n	Interleukin 1 receptor antagonist	0.55	n.s.	9.09
10938654	IL2rg	Interleukin 2 receptor, gamma	0.65	n.s.	4.34
10832306	Itgb2	Integrin, beta 2	0.65	n.s.	7.52
10907689	Itgb5	Integrin, alpha 5 (fibronectin receptor, alpha polypeptide)	0.60	n.s.	4.13
10907881	Mmp3	Matrix metallopeptidase 3	0.43	0.51	3.22
10780205	Mmp14	Matrix metallopeptidase 14 (membrane-inserted)	0.61	n.s.	2.66
10796476	Mrc1	Mannose receptor, C type 1	0.63	n.s.	4.64
10757898	Ncf1	Neutrophil cytosolic factor 1	0.60	n.s.	6.81
10764722	Ncf2	Neutrophil cytosolic factor 2	0.65	n.s.	4.32
10873336	Pla2g2a	Phospholipase A2, group IID	0.54	n.s.	4.76
10828351	RT1-Ba	RT1 class II, locus Ba	0.60	n.s.	3.92
10831557	RT1-Db1	RT1 class II, locus Db1	0.63	n.s.	2.50
10797509	Syk	Spleen tyrosine kinase	0.66	n.s.	4.14
**7 DAYS**					
10819379	Adh1c^a^	Alcohol dehydrogenase (class 1)	1.64	n.s	1.61
10854406	Akr1b10^a^	Aldo-ketoreductase family 1, member B10	1.90	n.s.	n.s.
10911380	Aldh1a2	Aldehyde dehydrogenase 1 family, member A2	1.81	n.s.	1.61
10722992	Anpep	Alanyl (membrane) aminopeptidase	2.44	n.s.	2.74
10744425	Alox15	Arachidonate 15-lipoxygenase	18.75	n.s.	n.s.
10719524		Apolipoprotein C-I (Apoc1)	1.69	n.s.	n.s.
10848030	Bbox1	Butyrobetaine (gamma), 2-oxoglutarate dioxygenase 1	n.s.	1.61	n.s.
10914614	Ccr2	Chemokine (C-C motif) receptor 2	n.s.	0.63	n.s.
10803991	Cd14	CD14 molecule	1.59	n.s.	2.06
10940473	Cd36	CD36 molecule (thrombospondin receptor)	3.97	n.s.	2.62
10935177	Cldn2	Claudin 2	3.27	3.85	0.19
10767373	Cxcr4	Chemokine (C-X-C motif) receptor 4	1.51	n.s.	n.s.
10936899	Cybb	Cytochrome b-245, beta polypeptide	1.65	n.s.	10.08
10765212	F5	Coagulation factor V (proaccelerin, labile factor)	2.22	3.03	0.46
10794734	F13a1	Coagulation factor XIII, A1 polypeptide	2.00	n.s.	n.s.
10810778	Dpep2	Dipeptidase 2	1.67	n.s.	1.84
10928761	Fn1	Fibronectin 1	2.37	n.s.	2.23
10939901	Gpr101	G protein-coupled receptor 101 (Gpr101)	n.s.	1.83	0.54
10804221	Gpr151	G protein-coupled receptor 151	n.s.	1.82	n.s.
10825915	Gstm2	Glutathione S-transferase mu 2	1.78	n.s.	n.s.
10806122	Hmox1	Hemeoxygenase (decycling) 1	1.98	n.s.	5.90
10937302	Htr2c	5-hydroxytryptamine (serotonin) receptor 2C		1.90	0.46
10841693	Lbp	Lipopolysaccharide binding protein	2.19	n.s.	1.94
10791250	Lpl	Lipoprotein lipase	1.55	1.71	0.56
10874918	*Ly96*	Lymphocyte antigen 96	1.51	n.s.	2.63
10859392	Mgst1	Microsomal glutathione S-transferase 1	1.67	n.s.	1.72
10842239	Mmp9	Matrix metallopeptidase 9	1.85	n.s.	n.s.
10907869	Mmp12	Matrix metallopeptidase 12	1.91	n.s.	5.62
10780205	Mmp14	Matrix metallopeptidase 14 (membrane-inserted)	1.60	n.s.	1.79
10893231	Mmp19	Matrix metallopeptidase 19	1.51	n.s.	2.91
10788427	Msr1^a^	Macrophage scavenger receptor 1	2.53	n.s.	3.36
10821698	Osmr	Oncostatin M receptor	1.51	n.s.	3.69
10860867	Pon1	Paraoxonase 1	3.55	3.53	n.s.
10860878	Pon3	Paraoxonase 3	1.79	n.s.	n.s.
10873341	Pla2g2a	Phospholipase A2, group IIA	1.61	n.s.	n.s.
10873336	Pla2g2d	Phospholipase A2, group IID	2.17	n.s.	n.s.
10751295	Pla1a	Phospholipase A1 member A	1.70	n.s.	1.68
10782271	Plau	Plasminogen activator, urokinase	1.70	n.s.	4.36
10823819	Rxfp1	Relaxin/insulin-like family peptide receptor 1	n.s.	0.62	n.s.
10912439	Rbp1	Retinol binding protein 1, cellular	1.81	n.s.	1.54
10761047	Serpine1	Serpin peptidase inhibitor, clade E, member 1	2.71	n.s.	n.s.
10819139	Tacr3	Tachykinin receptor 3	0.56	0.62	n.s.

a A rat gene symbol was not provided by the Affymetrix annotation file for this transcript, however, IPA identified a human ortholog. Therefore, the gene symbol for the human ortholog is listed here.

AT 7 d post-TBI, anakinra increased the expression of several receptors involved in signaling pathways including 5-hydroxytryptamine (serotonin) receptor 2c, cholinergic receptor, nicotinic, alpha 3, fibroblast growth factor, G-protein-coupled receptor 101 and 105 and prolactin receptor (Table 2). EPO treatment caused an over expression of genes involved in the coagulation system (coagulation factor V (F5), coagulation factor XIII, A1 polypeptide, plasminogen activator, urokinraase and serpin peptidase inhibitor, clade E) and expression of genes involved in acute phase response signaling (hemeoxygenase 1, fibronectin 1, oncostatin M receptor, lipopolysaccharide binding protein, serpin peptidase inhibitor member 1 and retinal binding protein 1) (Table 2).

**Figure 6:**
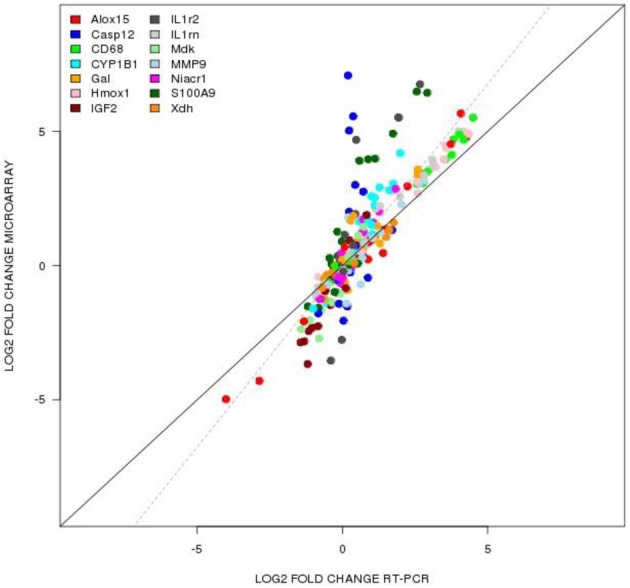
**TaqMan based RT-PCR validation of the microarray data for the selected genes: Alox15 (arachidonate 15-lipoxygenase), Casp12 (caspase 12), CD68 (CD 68 molecule), Cyp1b1 (cytochrome P450 1b1), Gal (galanin), Hmox1 (hemeoxygenase 1), IGF2 (insulin like growth factor 2), IL1rn (interleukin 1 receptor antagonist), Mdk (midkine), Mmp9 (matrix metallopeptidase 9), Niacr1 (niacin receptor 1), S100a9 (S100 calcium binding protein A9), Xdh (xanthine dehydrogenase)**. The RT-PCR data was normalized to the housekeeping gene β-actin. Pearson's coefficient, *r* = 0.859.

We used TaqMan-based quantitative-PCR (qPCR) analysis to validate gene expression changes of 14 genes (Alox15, Casp12, CD68, CYP1B1, GAL, HMOX1, IHF2, IL1r2, IL1rn, MDK, MMP9, NIACR1, S100A9, XDH) selected from specific pathways of interest in TBI. The data generated via microarray and quantitative-PCR (qPCR) was normalized to β-actin. Figure 6 shows that the qPCR findings were highly correlated with the microarray data (Slope = 1.352, Pearson's *R* = 0.859).

## Discussion

The dosage regiment used in our studies of anakinra and EPO were designed to produce concentrations in the range reported in patients receiving FDA approved doses and to reflect initiating therapy within 4 h of a head injury in patient. Clinically, administering an intravenous (i.v.) loading dose of either drug results in rapid peak concentrations, compared to the delay in peak found with our experimental model (i.p) initiated 2 h post-TBI. The FDA approved dosage recommendations for EPO is 150 U/kg administered s.c. three times weekly or 40,000 U weekly. EPO at a dose of 40,000 (~500 IU/kg) proposed for use in critically ill patients (Corwin et al., 2002) and TBI patients (Nirula et al., 2010) when administered i.v. would result in an peak concentration of approximately 10,000 mIU/ml with an average elimination half-life of 17 h and reaching non-detectable concentrations by 3 days post-dose (McCluskey et al., 2009). The recommended dose of anakinra in rheumatoid arthritis is 100 mg/day administered s.c. A pilot study of anakinra in acute stroke patients, receiving 100 mg i.v. bolus followed by a 2 mg/kg·h infusion resulted in median concentrations ranging from 20 to 40 μg/mL (Emsley et al., 2005).

The immunomodulation effects of EPO and anakinra did not translate into effects on functional behavioral and lesion studies. The severities of these deficits were similar to what we have observed using this model of injury previously (Kuypers and Hoane, 2010; Swan et al., 2011; Vonder Haar et al., 2011; Peterson et al., 2012). Treatment with either EPO or anakinra failed to induce significant beneficial effects on recovery of function in all four behavioral tests examined in this study. Additionally, both drugs failed to produce any significant effects on the prevention of injury induced tissue loss in the cortex or hippocampus when examined 30 days post-injury.

The use of microarray analysis to delineate gene expression patterns and profile changes is a powerful tool that can be used to evaluate the potential effect of treatment, in addition to increasing the understanding of the mechanism of the treatment effect. Both EPO and anakinra treatments penetrated the brain and altered the inflammatory and immune pathways.

The lack of effect of EPO and anakinra on functional recovery of cognition and motor behavior is in contrast to the significant effect of other drugs such as progesterone and nicotinamide when administered for 72 h in the same CCI model (Peterson et al., 2012). Both nicotinamide and progesterone treatments also resulted in significant effects on the expression of genes involved in the inflammatory/immune pathways (Anderson et al., 2011, 2013). However, in contrast to EPO and anakinra, both nicotinamide and progesterone also demonstrate significant effects on gene expression of other biological pathways (Anderson et al., 2011). Nicotinamide treatment resulted in the differential expression of genes involved in the inflammatory/immune pathways only at 24 h and not at 72 h and 7 days with IPA analysis also identified over-expression of genes involved in 1L-10 signaling, LXR-RXR activation, TREM1 signaling, communication between innate and adaptive immune cells and PPAR signaling. However, the canonical pathways identified at 72 h after injury for nicotinamide included a significant effect on cell signaling pathways involving neurotransmitters, neuropeptides, growth factors and ion channels (Anderson et al., 2013). In addition to positive and negative effects on inflammatory pathways, progesterone also affected genes involved in cell proliferation, DNA damage response, regulation of apoptosis and blood vessel remodeling (Anderson et al., 2011).

EPO significantly increased the gene expression of arachidonate 15-lipoxygenase (Alox15) at 24 h, 72 h and 7 days post-TBI and anakinra increased the expression of Alox15 at 24 and 72 h post-TBI. Alox15 is considered to have a pro-inflammatory effect and may play a key role in the acute inflammatory response and reportedly leads to the generation of unstable lipid products from arachidonate (Kuhn and O'Donnell, 2006). Previous studies investigating the effect of two different doses of progesterone (Anderson et al., 2011) and nicotinamide (Anderson et al., 2013) on gene expression in the CCI model found that neither nicotinamide infusion nor low dose progesterone increased Alox15. In contrast, the higher dose of progesterone did significantly increase gene expression of Alox15. When low and high dose progesterone and nicotinamide were compared on the functional recovery of cognitive behavior in the same model, the low dose progesterone and nicotinamide produced significant effects on functional recovery that were not found with the higher dose. Nicotinamide and low dose progesterone also reduced tissue loss compared to vehicle (Peterson et al., 2012). As high concentrations of IL-1ra are associated with better outcome in patients (Hutchinson et al., 2007), the decreased gene expression of IL-1rn by EPO at 24 and 72 h and by anakinra at 24 h post-TBI may also be a significant negative effect of the treatment. Both doses of progesterone increased IL-1rn at 72 h (Anderson et al., 2011). Nicotinamide did decrease gene expression of IL-1rn at 24 h with no effect at 72 h or 7 days post-injury (Anderson et al., 2013).

The majority of the pre-clinical research in animal models evaluating the effectiveness of EPO in brain injury utilized a dose of 5000 U/kg, which was tested at various time-points ranging from a pretreatment up to 9 h post-injury, with earlier administration resulting in greater benefits (Brines et al., 2000; Cerami et al., 2002). However, beneficial effects were found with administration as late as 24 h post-injury (Zhang et al., 2009; Meng et al., 2011) and with multiple dose studies administered up to 14 days (Lu et al., 2005; Liao et al., 2008; Xiong et al., 2008). Meng et al. found dose dependent effects of EPO when administered i.p. at 24, 48, and 72 h (Meng et al., 2011). All doses (1000–7000 U/kg) provided some neuroprotection in the form of less hippocampal cell loss and improved sensorimotor and spatial learning performance in comparison to the sham group (Meng et al., 2011). However, the optimal dose was 5000 U/kg for a significant improvement in both histological and functional outcomes. This dose would result in EPO concentration considerably above those associated with the current FDA recommendations (150 U/kg) (Rizzo et al., 2010).

This study is limited by evaluating only one dosage regiment initiated at one time point. Experimental studies have demonstrated both a neurotoxic as well as neuroprotective function of inflammatory response (Lenzlinger et al., 2001). The response is pro-inflammatory during the acute phase and anti-inflammatory during the chronic phase, which theoretically assists in repair and recovery processes(Correale and Villa, 2004). Therefore, the timing of the inflammatory modulator treatment may be critical. The limited pre-clinical data evaluating IL-1ra in TBI, suggests that initiating therapy later for anakinra would not increase efficacy, however, increasing the treatment duration could improve outcome. Treatment of IL-1ra, 100 mg/kg s.c. reduced injury volume by 44% when treated within 15 min, but only 28% when administrated at 4 h in the fluid percussion injury (FPI) model (Toulmond and Rothwell, 1995). In the FPI model, when IL-1ra 100 mg/kg was administered 15 min after injury and animals were treated for 7 days, IL-1ra significantly reduced neuronal loss in the hippocampus and the cortex. A lower dose (10 mg/kg) had no significant effects (Sanderson et al., 1999). As starting therapy within 15 min of a head injury is not feasible, if early initiation is necessary for effect, anakinra would not provide a practical treatment. For EPO, initiating therapy at 24 h post-TBI and treating for a long duration may have improved the effects.

Anakinra and EPO initiated at dosage regiments designed to produce concentrations in the range reported in patients receiving FDA approved doses did result in significant effects on gene expression in the brain reflecting adequate penetration and altered genes involved in inflammatory processes; however, the dose regiments were not sufficient to produce neurorestorative effects. This data suggests that targeting inflammatory processes alone may not be sufficient for preventing secondary injuries after TBI. Ultimately, a polytherapy approach that addresses the multitude of immediate and prolonged symptoms of TBI may be the key to an effective overall treatment.

## Author contributions

**Table:**
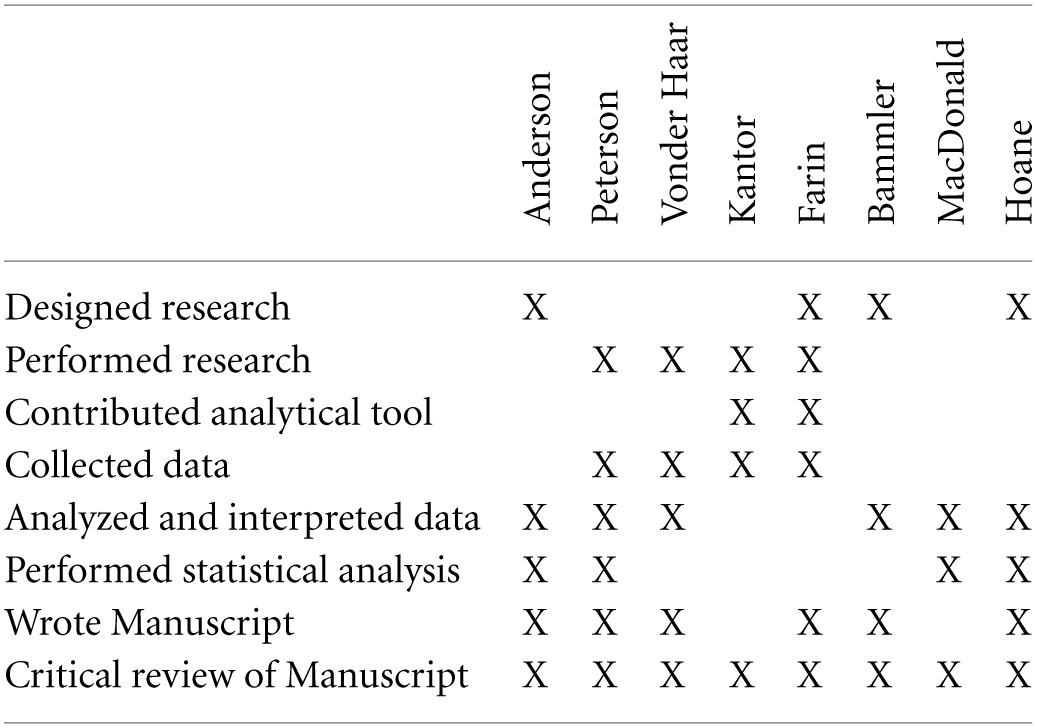

### Conflict of interest statement

The authors declare that the research was conducted in the absence of any commercial or financial relationships that could be construed as a potential conflict of interest.
